# Finite element simulation of the braiding process

**DOI:** 10.1186/s40759-019-0041-4

**Published:** 2019-01-23

**Authors:** S. Del Rosso, L. Iannucci, P.T. Curtis

**Affiliations:** Imperial College London, Exhibition Road, London, SW7 2AZ UK

**Keywords:** Braiding, Finite element

## Abstract

Braiding is one of the most common technique employed for the manufacture of fabrics and ropes. It is also commonly used to produce near-net shaped preforms for advanced fibre reinforced composites. This paper presents an explicit finite element approach to create and simulate the braiding process for the virtual manufacture of 2D braids. The process starts from the definition of an analytical function which describes the movement of the carriers on a braiding track plate. Models of idealised Maypole-type braiding machines are built and used to shape virtual yarns into braids. This procedure can be used in a parameter control fashion, to optimise or to create virtual braided structures, which can serve as input for other structural analyses. It is emphasised that multiple cylinders are required for the modelling of a multifilament yarn to achieve better correlation with the experimental results. A parametric study is presented to investigate the effect of the number of virtual cylinders to represent a real yarn and the shape of the final braid. Excellent correlation was found between the virtual models and the experimental results when comparing the braid angle and yarn width.

## Introduction

Computer simulations have been extensively used in engineering manufacture to understand each phase of the production and design process. Process simulations can be used to replace costly trial-and-error and product development steps to produce parts with optimal design, and tailored physical and mechanical properties. Many industrial processes, such as injection moulding, forming, stamping, welding, additive manufacturing, as well as automation and robotics rely on computer simulations (Radi and Hami 2016; Salvendy 2001). In the development process, the finite element (FE) method is also widely used to simulate manufacturing steps, and for the virtual predictions of mechanical properties of the final product. In the composite materials field, FE aids in the understanding and optimisation of individual manufacturing operations and analysis of the response of the final product during its service life. The so called FE method can be used to create and simulate the physical braiding process. It can also be used as an optimisation tool to determine the best braid geometry to fulfil the mechanical requirements in specific engineering applications.

The FE method has been used in different studies to shape virtual braids by starting from an arbitrary yarn configuration. For example, Durville et al. (2015) produced virtual braids starting from interpenetrating, helicoidally arranged filaments which were gradually separated at the cross-over points using contact-friction algorithms. Wehrkamp-Richter et al. (2018) used a multi-step approach to create undulated yarns which were then separated and compacted by “fictitious” thermal disturbance and pressure. Other authors (Shanahan et al. 2017; Peirlinck et al. 2018) used an in-house developed FE code to create braided stents starting from sets of arbitrary spatial coordinates and repeated in the 3D space. The approaches proposed in (Vu et al. 2015; Wehrkamp-Richter et al. 2018; Shanahan et al. 2017; Peirlinck et al. 2018) allow creation of virtual braids with geometries very close to their real counterpart. However, these processes start from a static initial geometry and then parameters such as braid angle, braid diameter and braid pitch are tuned until a geometrical correlation with the actual braid is found.

An alternative to this approach is to use the FE method to directly replicate the braiding process. For example, Pickett et al. (2009) investigated the effect of different machine settings in the braiding of carbon tows over an irregular-shape preform. Shell elements were used to model the tooling surfaces whilst bar and beam elements were used for bias and unidirectional axial yarns, respectively. Material properties such as friction coefficients for yarn-to-yarn contact and yarns-to-tooling contact, and yarn bending were included in the modelling. Good correlation was found with respect to the experimental observations. Van Ravenhorst and Akkerman (2014) proposed an inverse-kinematic solution for the modelling and simulation of a circular braiding machine. They simulated the braiding process over a mandrel with an irregular cross-section and centre of symmetry. Models showed good correlation with experimental results, although the difference in braid angle was as high as 10 ^∘^ in certain regions of the overbraided mandrel. The assumptions made, such as no yarn interaction, no friction between yarns and other machine parts, zero yarn thickness and immobility of the yarns after being deposited, are deemed to be the cause of mismatch between prediction and experimental observations. As described in (Goyal et al. 2005; Pickett et al. 2009), it is challenging to capture the effective lenticular cross-sectional geometry of the bias yarns. The use of beam elements with a circular cross-section could lead to model inaccuracies at micro-scale level. For example, the lenticular shape the yarn adopts within a braid cannot be captured with such elements. Large errors, especially in the calculation of the transverse properties of the braid, may occur when the lenticular yarn shape is not properly modelled. Wang and Sun (2001); Wang et al. (2004, 2010) introduced the concept of “digital-elements” for the virtual manufacture of textile fabrics. Filaments in a yarn are discretised as a sequence of rod-elements connected by frictionless pins. This approach allows the simulation of a variety of textile manufacturing processes such as weaving or braiding, and investigation of the shape that the filaments assume during the conversion into a textile. Numerical results matched well with experimental observations.

In this paper, we present a simple technique to replicate the braiding process for the manufacture of virtual braids. The technique allows for the creation of circular Maypole braiders with an arbitrary number of horn gears, carriers and dimensions. It will also be emphasised that the use of a single cylinder to model a multifilament yarn cannot accurately represent the yarns once braided. A parametric study on the effect of the number of virtual cylinders used to model a real yarn is presented. Comparison of the shape of the virtual braids with experimental results are also shown.

## Methods

### The braiding process

**Fig. 1:**
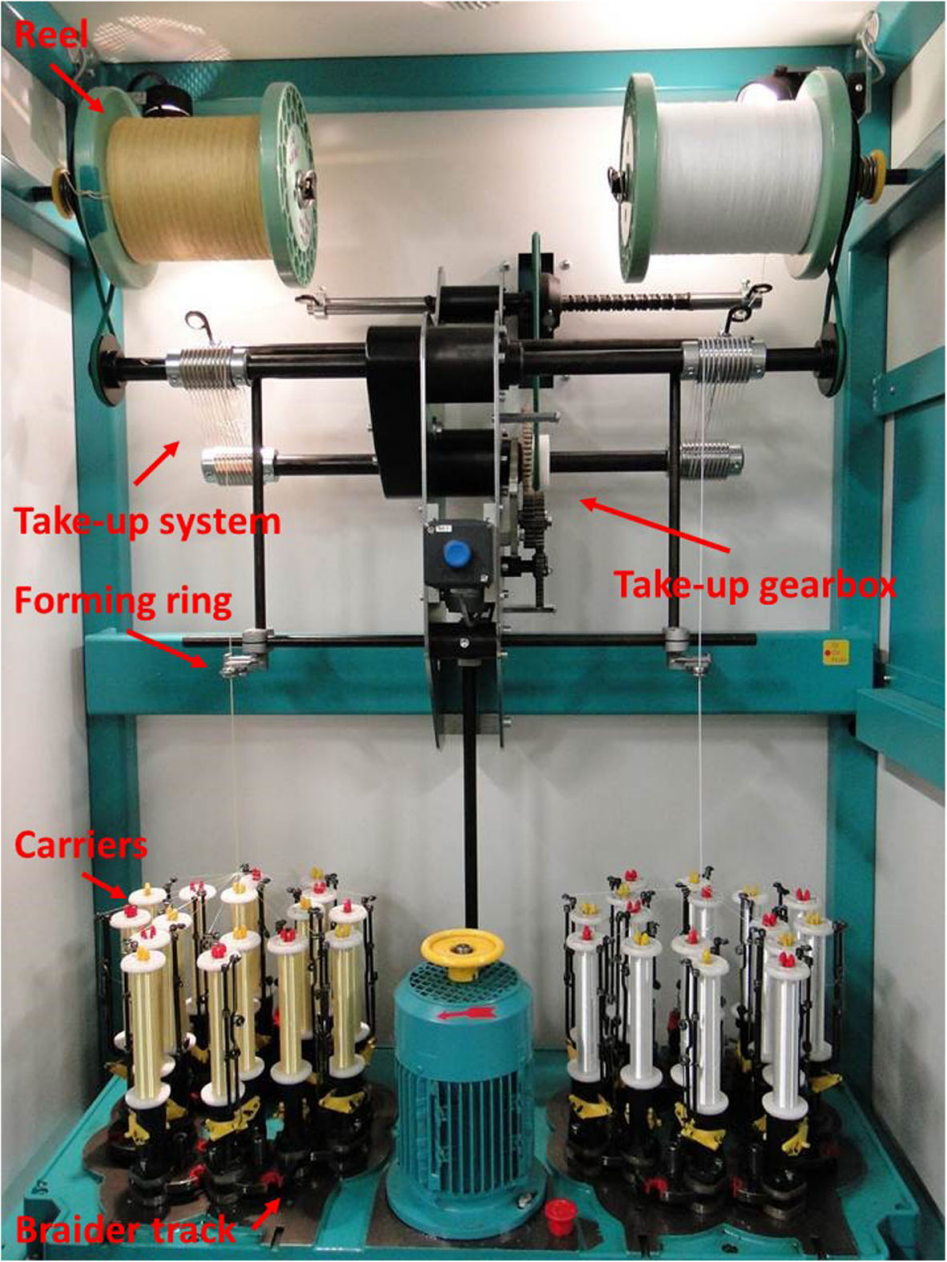
Maypole-type braiding machine

Braiding is the process of interlacing three or more strands diagonally to form a regular and ordered structure. The most common type of braiding machine is the “Maypole” braider, as depicted in Fig. 1. This can be either horizontally or vertically mounted.

**Fig. 2:**
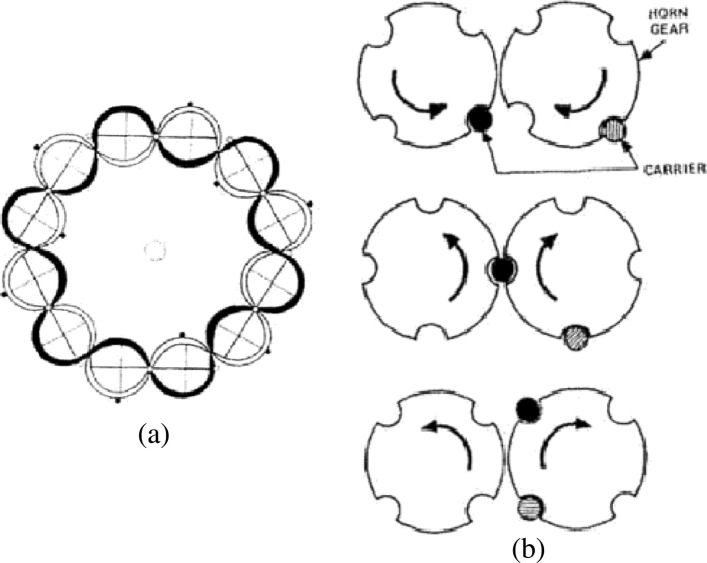
(**a**): Carrier path on the braider track plate (reproduced from (Lee 1992)); (**b**): Horn gear mechanism (reproduced from (Lee 1992))

Yarns are rewound to bobbins or spools which are then fitted on carriers. Carriers are moved in a pseudo-sinusoidal fashion on the braider track plate by horn gears (Fig. 2a). Half of the carriers move clockwise and the other half move in the opposite direction. When a carrier meets the oncoming horn gear, it switches to the latter by a mechanism similar to a railway switch (Fig. 2b). Carriers are also equipped with a tension adjustment spring system which keeps the yarn uniformly in tension while it is driven along its path, preventing slack or loosened fibres. Carriers move so that yarns intertwine in a pre-determined fashion creating the desired braid architecture. The intertwining yarns converge, tighten up and pass through a forming ring. The take-up system withdraws the braid at constant speed while forming, and it is finally wound up onto a reel. The braid angle is defined as the angle between the bias yarn and the take-up direction.

### Definition of the carriers’ path

An analytical function which describes the paths of the carriers on the braiding track was derived. This function defines the instantaneous position of the carriers during the braiding process, and can be successfully applied for any Maypole-type braider with an even number of horn gears. Only two parameters must be defined: *N*_*h*_ and *r*_*c*_, which are the number of horn gears and the radius of the horn gears, respectively.

**Fig. 3:**
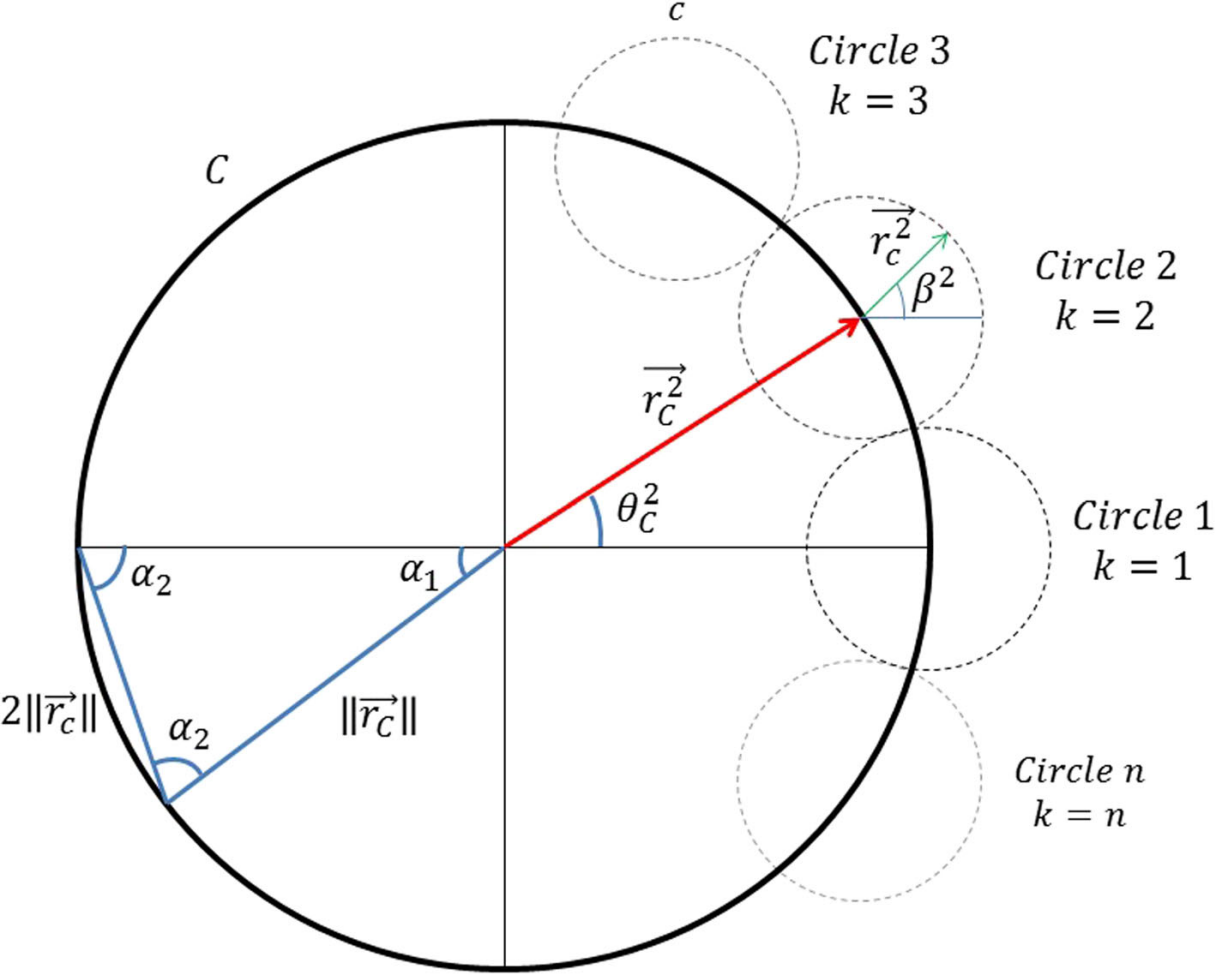
Definition of the carriers path on a braiding track plate

Consider the circle $\mathcal {{C}}$ of radius $r_{\mathcal {C}}$ in Fig. 3. $\overrightarrow {{r_{\mathcal {C}}^{k}}}$ is the vector $\overrightarrow {r_{\mathcal {C}}}$ from the centre of the circle $\mathcal {C}$ to the centre of the *k*^*th*^ circle $\mathcal {C}$. $\overrightarrow {{r_{\mathcal {C}}^{k}}}$ is a vector equal in magnitude to the radius of the smaller circles $\mathcal {C}$ starting from the centre of the circle $\mathcal {c}^{k}$.

From the sine law we have:

1 $$ {\frac{\left\Vert\overrightarrow{{r_{\mathcal{C}}}}\right\Vert}{\sin \alpha_{2}}=\frac{2\left\Vert\overrightarrow{{r_{\mathcal{c}}}}\right\Vert}{\sin \alpha_{1}}}  $$

where the angles *α*_1_ and *α*_2_ are defined as:

2a $$\begin{array}{*{20}l} \alpha_{1} = \frac{2\pi}{N_{h}} \end{array} $$

2b $$\begin{array}{*{20}l} \alpha_{2} = \frac{(\pi-\alpha_{1})}{2} \end{array} $$

Substituting Eq. 2a and into Eq. 1 we have:

3 $$ \overrightarrow{\big\Vert{r_{\mathcal{C}}}}\big\Vert = r_{\mathcal{C}} = \frac{r_{\mathcal{c}} \sin \alpha_{2}}{2 \sin \alpha_{1}}  $$

Equation 3 defines the relation between $r_{\mathcal {C}}$ and $r_{\mathcal {C}}$ which represent the radius of the braiding track plate and the radius of the horn gears, respectively. The base vector $\overrightarrow {{r_{\mathcal {C}}}}$ and the local vector $\overrightarrow {{r_{\mathcal {C}}}}$ can be calculated as follows.

For *k*=1→*N*_*h*_

4 $$ {\theta_{0}}^{k}= (k-1)\alpha_{1}  $$

5 $$ \overrightarrow{{r_{\mathcal{C}}^{k}}} = \left|\begin{array}{l} \cos {\theta_{\mathcal{C}}^{k}}\\ [3pt] \sin {\theta_{\mathcal{C}}^{k}} \end{array}\right| r_{\mathcal{C}}  $$

The local vector $\overrightarrow {r_{\mathcal {c}}^{k}}$ is:

6 $$ \overrightarrow{r_{\mathcal{c}}^{k}} = \left|\begin{array}{l} \cos {\beta^{k}}\\ \sin {\beta^{k}} \end{array}\right| r_{\mathcal{c}}  $$

where *β* is the internal angle of the *k*^*th*^ circle.

The angle *β* traces the path of the function and changes rotational direction amongst the small circles.

7 $$ \beta^{k}=(-1)^{k+1}\varphi^{k}+\alpha_{2}-\pi+(k-1)(\pi-2\alpha_{2})  $$

where the ranges of *φ*^*k*^ are:

For *k*=1,3,5...

8 $$ \varphi^{k}=[0,(2\pi-2\alpha_{2})]  $$

For *k*=2,4,6...

9 $$ \varphi^{k}=[0,(2\alpha_{2})]  $$

Thus, any point on the path can be traced by Eq. 10:

10 $$ \overrightarrow{{r}^{k}}=\overrightarrow{{r_{\mathcal{C}}^{k}}}+\overrightarrow{{r_{\mathcal{c}}^{k}}}  $$

In order to define the vector travelling along the opposite path and in the opposite direction, one should invert both the ranges and definition of *φ*^*k*^ in Eqs. 8 and 9 as follows:

For *k*=1,3,5...

11 $$ \varphi^{k}=[(2\alpha_{2}),0]  $$

For *k*=2,4,6...

12 $$ \varphi^{k}=[(2\pi-2\alpha_{2}),0]  $$

**Fig. 4:**
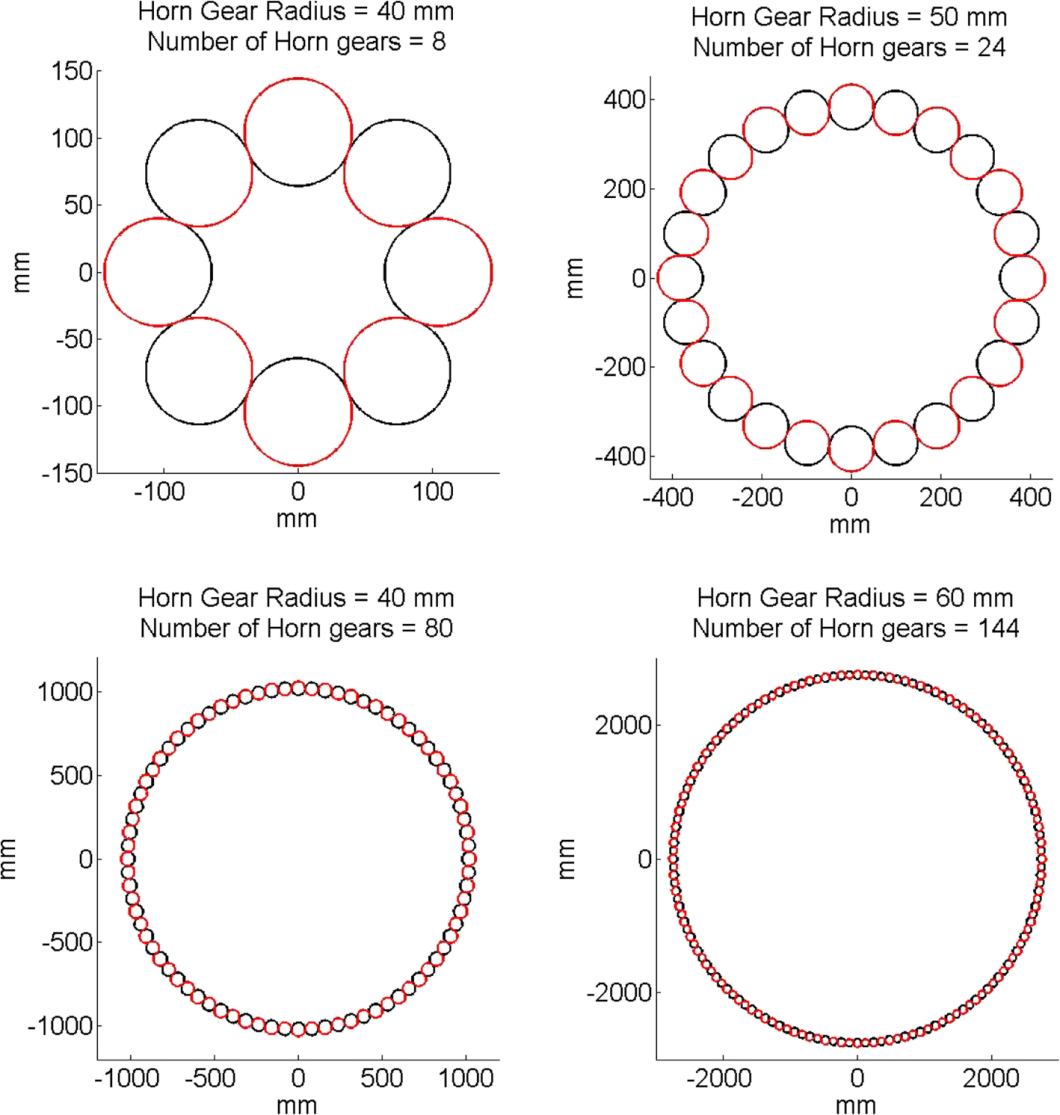
Carriers path definition for different braiding machines

This model can be successfully applied for the dimensional design of any Maypole-type braiding machine having an even number of horn gears. Figure 4 shows the paths of the carriers in four different Maypole type braiding machines. The position of the carriers along the braider’s plate can be tracked instantaneously using Eq. 10.

### Creation of the braiding machine using FE

**Fig. 5:**
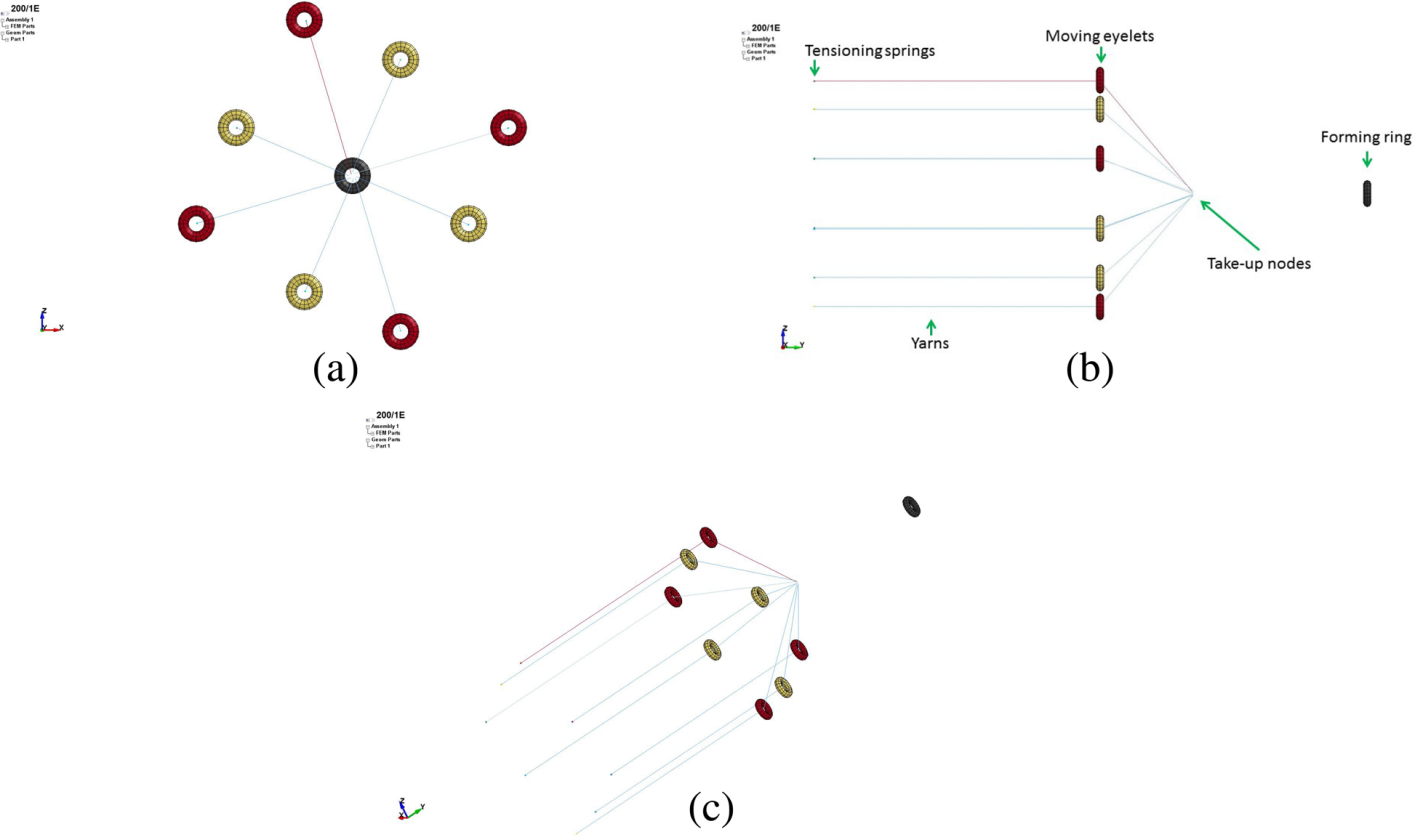
FE model of the braiding process: (**a**): Top view; (**b**): Side view; (**c**): Angle view

The 3D models of an idealised braiding machine were created using LS-PrePost, a LSTC pre- and post-processing software. The model consisted of different yarns, tensioning springs and eyelets corresponding to the number of yarns to be braided, and a forming ring. Figure 5 shows different views of the FE model for a braiding machine set up for the virtual manufacture of diamond 8 yarn braids.

The eyelets in the model represent the uppermost eyelet of each carrier whilst the forming ring was modelled as a fully constrained part. The normal distance of the moving eyelets to the fixed eyelet was constant for all simulations. All the eyelets and the forming ring had an inner radius of 7 mm and were modelled using solid elements with a minimum element length of 1.8 mm at its inner diameter. The yarns were 700 mm long and modelled with beam elements having a length of 1.5 mm. The beams were modelled using the Hughes-Liu element formulation (Hughes and Liu 1981a;), with a 2x2 Gauss quadrature. Although this setup would introduce bending stiffness to the beam elements, the length-to-diameter ratio would make the bending stiffness very small with respect to the axial stiffness. Moreover, this approach resulted in more robust handling of beam-to-beam and beam-to-solid contact compared to using the same beams with only one integration point through the thickness. The aspect ratio between different types of elements close to one was found the best in dealing with the contact between yarn and eyelet during the braiding process. Yarn-to-yarn and yarn-to-eyelet contact were defined among all parts in the LS-Dyna contact card. The static (*FS*) and dynamic (*FD*) coefficient of friction amongst the yarns were defined as 0.3 and 0.2, respectively, whilst the *FS* and *FD* among the yarns and the eyelets were both 0.1.

In a real braiding process, the natural forming point occurs somewhere between the forming ring and the carriers. This position depends on parameters such as take-up speed, yarn fineness and yarn-to-yarn coefficient of friction. The smaller the take-up speed, the higher the coefficient of friction and the coarser the yarn, the closer the natural forming point will be to the carriers. In the FE model, the yarns were modelled as converging to the center of the braiding machine and, when the simulation started, they were pulled from the take-up node (Fig. 5b) in the positive y-direction through the forming ring at a speed equal to the real braiding machine take-up speed *υ*_*tu*_. Based on experimental observations, the position of the take-up nodes relative to the carriers was adjusted depending on the take-up speed to be as close as possible to where the braid would form in a real braiding process. Opposite to the take-up nodes, linear elastic springs modelled with discrete elements, were attached to each yarn end to simulate the carrier tensioning springs (0.2 N was the effective pre-tension applied by the springs to the attached yarns). These springs, as in a real carrier, were restrained to move in the y-direction during their movement on the x-z plane, and were used to maintain a uniform tension in the yarns throughout the braiding simulation. The tension of the yarns at the beginning of the virtual braiding process was not uniform among different yarns. This is because of the difference in the yarn length and the relative position of the yarns with respect to the forming point. In order to even out the tension in each yarn before the starting of the braiding process, the springs were pulled in the negative y-direction for two seconds while keeping the uppermost nodes fully constrained. From their initial position, the eyelets and the yarn ends with springs were moved along a prescribed path established by Eq. 10 on the x-z plane. Their velocity was equal to the maximum carriers’ velocity of the braiding machine used for the manufacture of microbraids.

The number of elements in the models ranged between 19072 and 120544, depending on the number of cylinders used to model a real yarn. The simulation timestep was set constant to 1.1 10 ^−4^ s by adding non-physical masses only to elements which timestep would be less than the set one. In doing so, the stability of the simulations was unaffected and also the kinetic energy was approximately the same as if mass was not added. The virtual simulation of 20 seconds of the braiding process took between 23 and 114 hours with eight CPUs.

Both beam and solid elements were modelled using linear elastic material models. For the beam elements, The Young’s modulus *E*= 130 GPa and the density *ρ*= 1.44 g/cm^3^, which reflect the elastic material properties of Kevlar ^*Ⓡ*^49. The material constants used for modelling the eyelets were *E*= 220 GPa and *ρ*= 4 g/cm^3^. Tensioning springs were modelled using discrete elements.

**Fig. 6:**
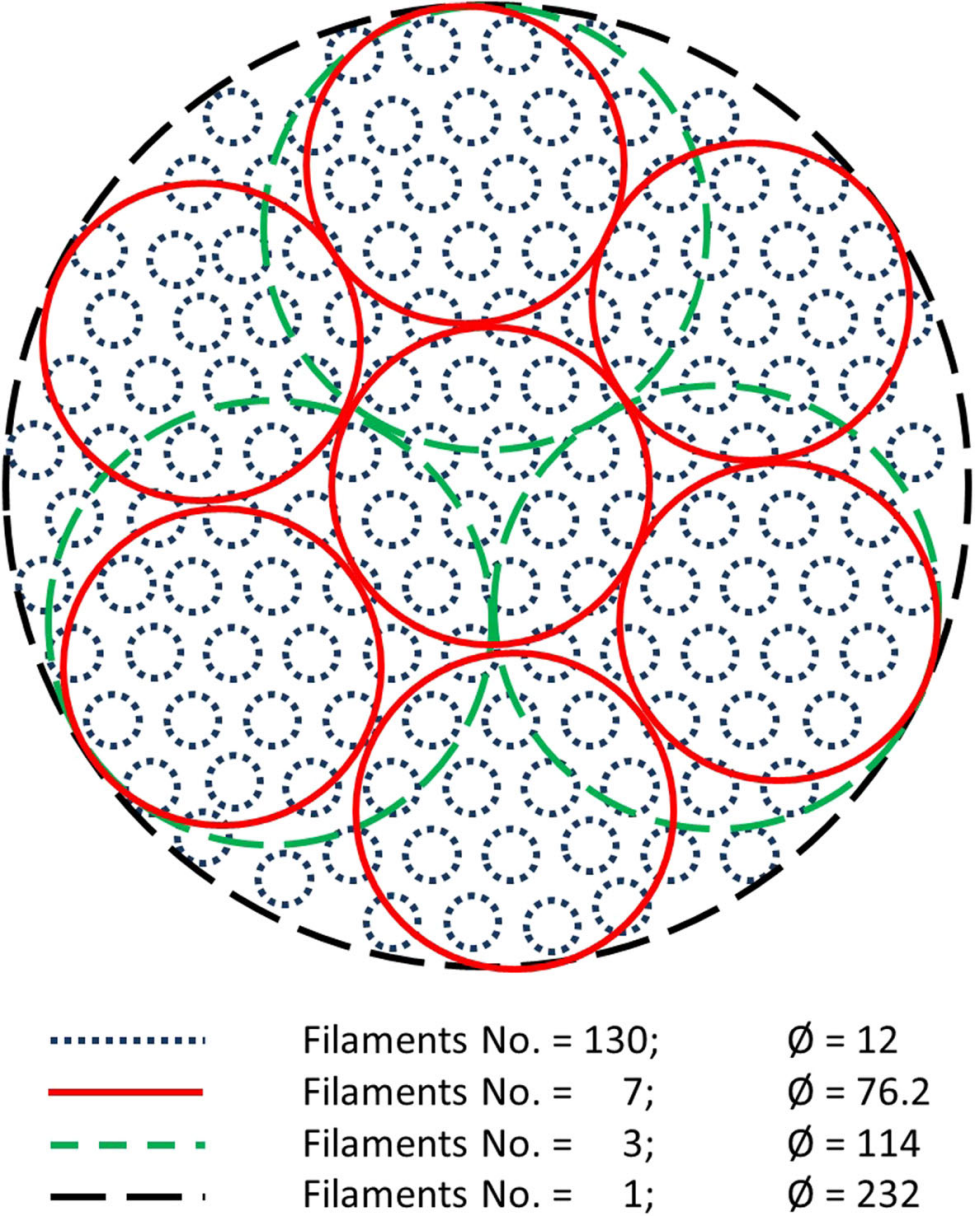
Cross-section of an idealised yarn

As discussed earlier, it may not be fully correct to model a multifilament yarn with a single cylinder. This is because the constitutive filaments of a real yarn change position and rearrange themselves during the forming process. Hence, in order to investigate to what extent the number of cylinders used to model a yarn affects the final shape of the virtual braid, a parametric study was carried out by using 1, 3 and 7 cylinders to model a multifilament yarn. The diameter of the cylinders was determined by defining a circle that encompassed the cross-sections of each of the individual filaments in a yarn with one large circle (Fig. 6). This large circle was then subdivided into 3 and 7 smaller circles. In order to shape braids with different braid angles, five different take-up speeds (510, 382, 255, 127 and 63 mm/min) were used.

**Fig. 7:**
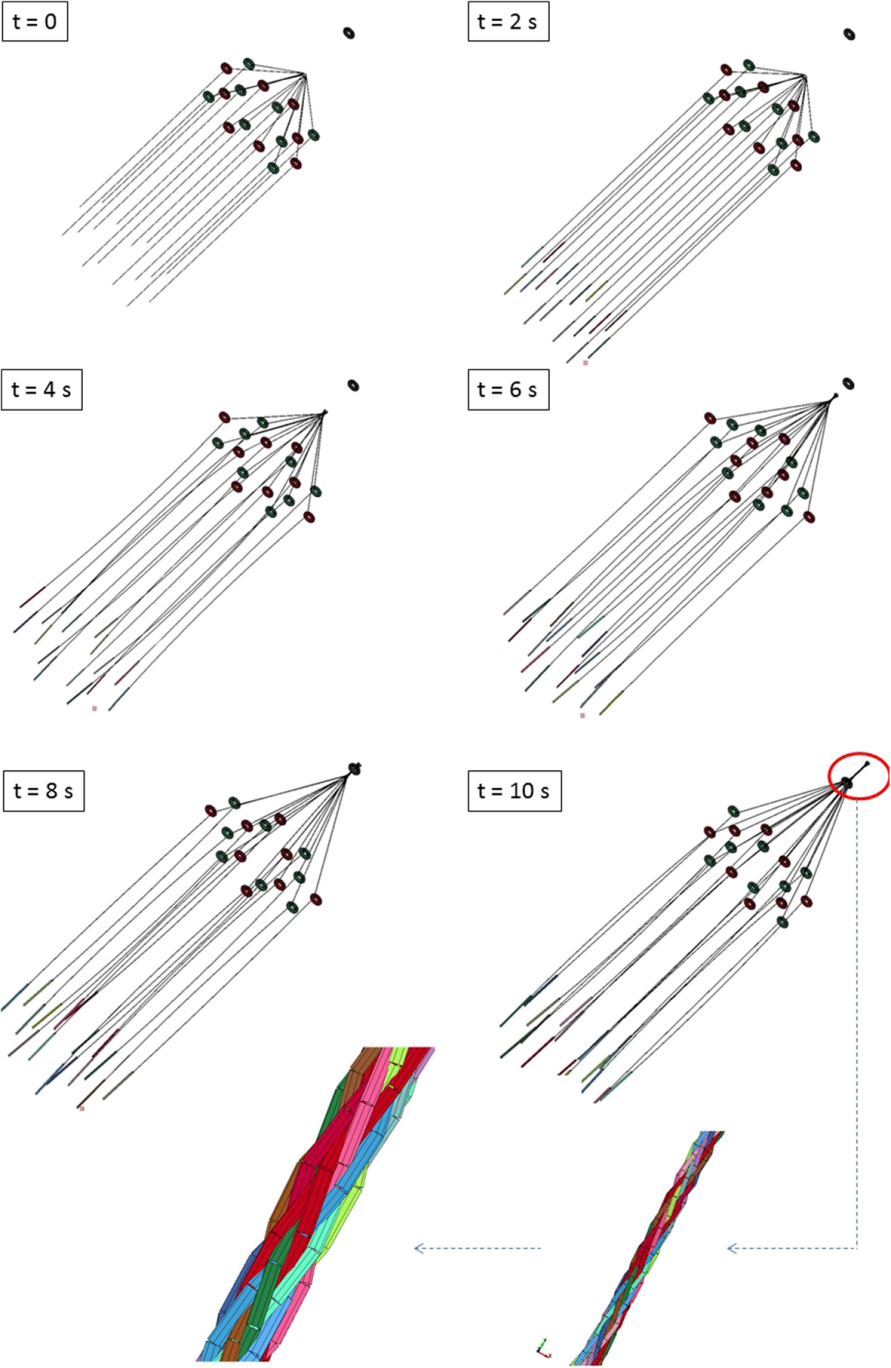
Simulation of the braiding process

Figure 7 shows a series of snapshots taken every 2 s of a typical braiding process simulation in which 16 threads were braided in a regular fashion.

## Results and discussion

### Effect of the number of beams on virtual braid geometry

**Fig. 8:**
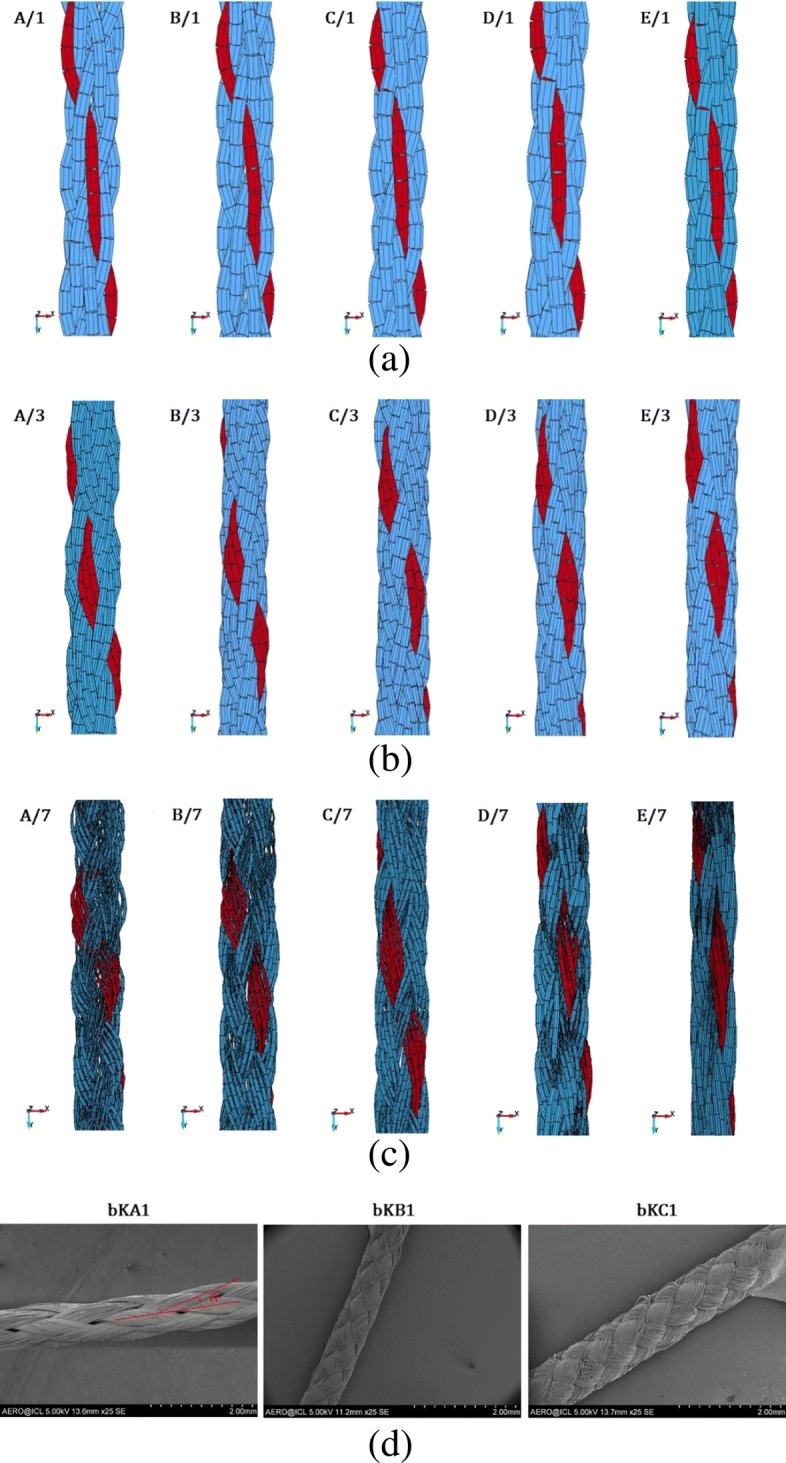
Snapshots of virtual braids created with different number of cylinders to simulate a multifilament yarn: **a**: One cylinder; **b**: Three cylinders; **c**: Seven cylinders; **d**: SEM images of Kevlar ^*Ⓡ*^49 microbraids

**Fig. 9:**
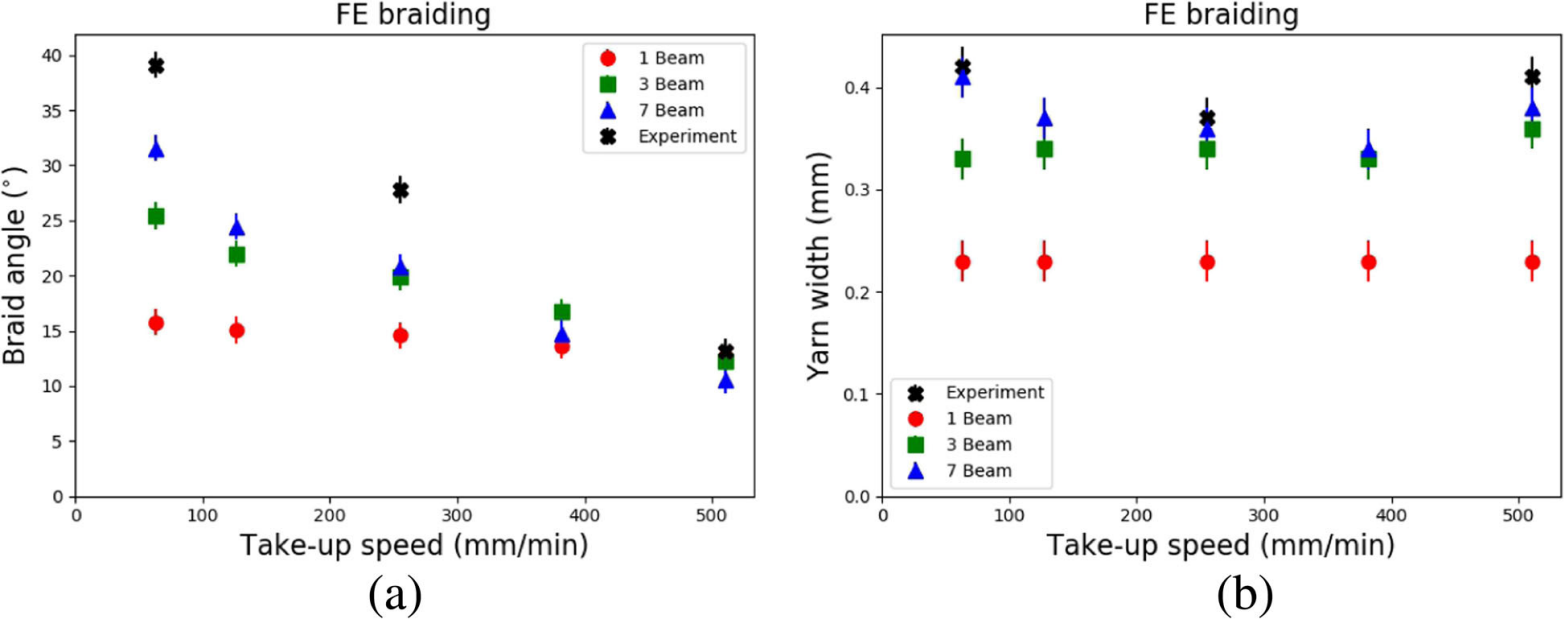
Comparison between FE models and experimental results (Del Rosso et al. 2015). **a**: Braid angle vs. take-up speed; **b**: Yarn width vs. take-up speed

Figure 8 shows the snapshots of different virtual braids created by interlacing eight threads in a diamond fashion. Five different take-up speeds *υ*_*tu*_ were analysed for each type of model. The lowest take-up speed was applied to model “A” whilst the fastest *υ*_*tu*_ to model “E”. One thread was coloured differently for visualisation purposes. Geometrical features of the resulting virtual braids, such as braid angle and yarn width, are compared with those of Kevlar ^*Ⓡ*^49 microbraids bKA1, bKB1 and bKC1, which were published in the author’s previous work (Del Rosso et al. 2015). Microbraids bKA1, bKB1 and bKC1 were manufactured using take-up speeds equal to 510, 255 and 63 mm/min, respectively. Results are graphically shown in Fig. 9.

It can be seen that the take-up speed affected the final shape of the braid. The higher *υ*_*tu*_, the lower the braid angle. However, when using only one cylinder to model a multifilament yarn, it appears that *α* was only slightly affected by the take-up speed. Although the difference in braid angle between model “E/1” and the real braid was negligible, the difference in *α* for the lowest take-up speed was as high as 23.3 ^∘^. Increasing the number of cylinders from one to three, an improved correlation was seen between the simulations and the experimental results. For the smallest *υ*_*tu*_, the braid angle determined for model “A/3” was 25.43 ^∘^. Although the difference in braid angle between the virtual and the real braid was as high as 13.67 ^∘^, a noticeable improvement with respect to the previous case was achieved as far as the braid angle is concerned. Increasing the take-up speed, the difference between the calculated braid angle for virtual braids and the braid angle of a real braid decreased. On the other hand, it must be emphasised that the running time of X/3 simulations was more than double the time required to complete the X/1 simulations (23 h compared to 51 h). Increasing the number of cylinders used to model a multifilament yarn to seven, the computational time increased to 113 h. Although the use of seven cylinders to model the real yarn did not lead to a significant difference in terms of braid angle with respect to the previous X/1 and X/3 models for high take-up speeds, a better correlation between the simulations and the experimental results was found for small *υ*_*tu*_. The difference in the braid angle between the virtual and the real braid was as low as 7.54 ^∘^ for *υ*_*tu*_= 63 mm/min. The apparent irregular braid shapes for models X/7 are due solely to the bad rendering of the prisms shaped around the beam elements (Fig. 8c).

In terms of yarn width *w*_*d*_, which is the width of the beams normal to the bias direction, it is possible to see from Fig. 8a that the width of the yarns of the virtual braids X/1 was the same for all models, regardless of take-up speed, and equal to the diameter of the cylinder beams. The cylinder beams were not able to flatten during the virtual braiding process, creating highly crimped structures regardless of the take-up speed. The difference in *w*_*d*_ between the real and the virtual microbraids was as high as 82.6%. Increasing the number of threads to three or seven, the shapes of the virtual microbraids were closer to their real counterparts, as observed in Fig. 8b and c. During the virtual braiding process, the beam cylinders were able to reorganise themselves and assume a position similar to the filaments in a real microbraid. The difference in yarn width between the real and virtual microbraids was as much as 27.2% for A/3 model and 7.9% for E/7 model, respectively. Figure 9b plots *w*_*d*_ vs. *υ*_*tu*_ for the created virtual braids.

From this study, it appears that modelling a multifilament yarn with a single cylinder (of solid or beam elements) is not the optimum method for the creation of virtual braid models. Although computationally cost-efficient, this method led to a large mismatch between the simulation and the experimental outcomes as far as the braid angle and yarn width are concerned. The use of three or more cylinders to model a single thread enhanced the quality of the shaped braid, however at the cost of a higher computational time. Nevertheless, the FE results only partially matched the experimental results. The possible causes of discrepancy between the experimental and FE results are associated with the simple elastic material model used for the yarns, and parameters such as yarn tension, yarn-to-yarn and yarn-to-eyelet coefficient of friction. In order to reduce the computational time when simulating multifilament yarns, the discrete element method (DEM) could be used to create on-the-fly beam elements during the virtual braiding simulation.

## Conclusion

This study has shown that the Finite Element technique can be successfully applied to replicate the braiding process for the creation of virtual braids. The proposed method can be easily implemented to replicate the behaviour of any Maypole type braiding machine for the manufacture of braids of different architectures, number of braided threads and braid patterns. Moreover, this technique can be employed for the creation of virtual braids with different materials if realistic material properties are used. It has been shown that the use of single cylinders made of beam elements can be successfully employed for the virtual manufacture of open-mesh braids. However, as shown, it is not sufficient to model a multifilament yarn with a single cylinder. In order to correctly capture geometrical features of real braids made of multifilament yarns, the use of three or more cylinders to model a thread is highly recommended for the FE simulations, although at a higher computational cost.

## Abbreviations

FE: Finite element
